# Clinical outcomes of simultaneous pancreas-kidney transplantation in elderly type II diabetic recipients

**DOI:** 10.1186/s13098-024-01295-y

**Published:** 2024-02-29

**Authors:** Yu Cao, Jie Zhao, Gang Feng, Zhen Wang, Jianghao Wei, Yang Xu, Chunbai Mo, Wenli Song

**Affiliations:** Department of Kidney transplantation, Tianjin First Central Hospital, Nankai District, 300192 Tianjin, China

**Keywords:** Simultaneous pancreas and kidney transplantation, Type II diabetes mellitus, Age, Survival outcomes, Metabolic outcome

## Abstract

The effect of age on outcomes after simultaneous pancreas-kidney transplantation (SPK) among type II diabetes (T2DM) recipients remains inconclusive. This study aimed to analyze the relationship between the age at time of transplantation and mortality, graft loss and metabolic profiles of T2DM SPK recipients. A retrospective cohort consisting of T2MD SPK recipients in a single transplant center was established. The baseline clinical characteristics and outcomes were collected and analyzed based on the age groups divided by 55-year-old. Time-to-event data analysis was performed using Kaplan-Meier method, and competing risk method was adopted to calculate the cumulative incidence of graft loss. A mixed regression model was applied to compare metabolic outcomes including glycated hemoglobin (HbA1c), fasting blood glucose, triglyceride, cholesterol, low-density lipoprotein, and higher estimated glomerular filtration rate (eGFR). 103 T2DM SPK recipients were included, of which 35 were > = 55 years old and 68 were < 55 years old. Baseline characteristics were comparable between age groups. The results indicated that comparable 5-year survival outcomes between groups with functioning grafts perioperatively. Additionally, no relationship of age with graft loss, complications and metabolic outcomes was detected.

## Introduction

Diabetes is a global burden of disease that is globally growing at a remarkable rate. There were more than 536 million people diagnosed with diabetes mellitus (DM) worldwide in 2021 [1] Type 2 diabetes (T2DM) is the most common type of diabetes, accounting for over 90% of all diabetes worldwide [2]. The global prevalence of Type 2 diabetes was 6059 cases per 100,000, with roughly 462 million individuals being affected by Type 2 diabetes, equivalent to 6.28% of the global population [3]. The 2021 International Diabetes Federation Diabetes Atlas Report reported that China has the most people with diabetes with estimates of over 140 million in 2021, reaching over 174 million by 2045 [1]. Furthermore, diabetes is still the main cause of end-stage kidney disease worldwide [4, 5].

Lately, the first world consensus conference suggested that in suitable T2DM recipients, SPK transplantation improves quality of life and survival compared with patients remaining on dialysis or deceased donor kidney transplantation alone as well [6]. Moreover, the advances in diabetes care, like better management of insulin therapy, improved self-control of blood glucose levels, organ-protective and antiproteinuric medications, have allowed more people with T2DM to live longer [7, 8]. Accordingly, an increasing number of aging patients with DM were referred for transplantation [9]. Though there was a growing body of studies evaluating outcomes of SPK in older patients with DM, majority of the subjects were T1DM recipients [10–17]. Research on T2DM patients who traditionally had higher BMI, were older and had longer duration of pre-transplant dialysis along with more intense comorbidities, were limited [18–21]. The purpose of this study was to evaluate the impact of age on survival outcomes and metabolic outcomes among T2DM SPK recipients.

## Patients and methods

### Study population and data collection

Transplantations for T2DM patients with ESRD in the Tianjin First Central Hospital from January 2015 to November 2021 were retrospectively analyzed, and after excluding 27 subjects below 18 years old or non-primary transplantation, 103 SPK patients were included. The follow-up of this cohort ended in Nov 2022 and all participants were followed for at least 1 year. All organs were obtained from deceased donors (DD), and no donor was a prisoner at the time of organ procurement [22, 23]. The medical records were obtained from the electronic medical documentation system. Follow-up information was from the medical records system or phone contact. The study was approved by the ethnicity committee of the hospital (NO. 2023DZX16).

### Baseline characteristics and comorbidities

Baseline clinical characteristics and comorbidities before transplantation were recorded at the time point of transplantation which included age, sex, body mass index (BMI) of recipients’ and donors’, dialysis, immunosuppressive induction and maintaining drugs, cardiovascular disease before and after transplantation, cerebral diseases before and after transplantation and panel reactive antibody (PRA). The T2DM definition was based on the 1999 WHO guideline and 2013 Guidelines for the prevention and control of type 2 diabetes in China [24, 25]. The selection criteria of SPK candidates were based on the Chinese Pancreas Transplantation Guideline [26]. For the PRA result, if the percent of panel reactive antibody was > 10%, the PRA result was positive, otherwise the PRA result was negative; Pre-transplantation comorbidities included cardiovascular diseases and cerebrovascular diseases. Cardiovascular diseases were identified if there were previous myocardial infarction and previous coronary intervention documented in the records. Patients with cerebrovascular diseases had documented transient ischemic attack (TIA), ischemic stroke or cerebral hemorrhage in their case histories. For those above 50 years old, coronary artery CT scan would be administrated for evaluation before transplantation.

### Outcomes

The primary outcomes were recipients’ and grafts’ survival rates. Death was defined as mortality from any causes. Renal graft failure was defined as patient death, kidney re-transplantation and returning to dialysis. Pancreas graft failure was defined as resumption of daily scheduled insulin, allograft pancreatectomy and patient death. Renal function was evaluated by estimated Glomerular Filtration Rate (eGFR), calculated based on Modification of Diet in Renal Disease Study Equation (MDRD). Metabolic outcomes included glycated hemoglobin (HbA1c), fasting blood glucose, triglyceride, cholesterol, low-density lipoprotein, and eGFR. Complications covered rejection, infection, re-administration, reoperation, cardiovascular disease, cerebrovascular disease. Kidney rejection was biopsy diagnosed. Pancreas rejection relies on clinical signs as well as laboratory evidence of elevated serum amylase, lipase, and glucose. DGF was diagnosed as returning to dialysis within 7 days after transplantation.

### Immunosuppression

Anti-thymocyte globulin or anti-IL-2R monoclonal antibody were given as induction to patients during the operation. The patients were treated with a triple immunosuppressive regimen post-transplant that consisted of tacrolimus (or cyclosporine A as an alternative), mycophenolic acid (MPA) (including mycophenolate mofetil or enteric-coated mycophenolate sodium), and steroids.

### Statistical analysis

All statistical analyses were performed using R studio (RStudio 2022.07.1 + 554 “Spotted Wakerobin” Release). Descriptive statistics were used to report the demographic characteristics. Absolute (n) and percentage (%) values were used for categorical variables. The numerical variables were reported according to its distribution using mean and standard deviation for normally distributed variables, and median and inter-quartile range (IQR) for non-normally distributed variables. Normally distributed variables were expressed as the mean ± standard deviation (SD) and were compared with Student’s t-test. Non-normally data were compared using the Wilcoxon signed-rank test. Categorical variables were compared using Chi-squared test or Fisher’s test.

The overall survival of recipients was estimated with Kaplan-Meier (KM) method and the comparison between groups was done by log-rank test. Considering the fact that survival benefit of SPK depending on the successful early graft function [27], the subgroup analysis for patients with functioning graft during peri-operative period was conducted. The cumulative incidence of graft loss was calculated by Competing Risk Analysis method (CRA) using cumulative incidence function (CIF) where mortality was treated as a competing risk with graft loss. The cumulative incidence was evaluated using the Aalen-Johansen estimator [28], with differences being tested using Gray’s tests [29]. A 2-sided *P*-value < 0.05 was considered to be statistically significant.

## Results

### Patient characteristics

103 recipients consisted of 91 males and 12 females, with a mean age of 50.3 ± 9.2 years (maximum age of 72.8 years old, minimum of 27.1 years old) and a mean BMI of 24.7 ± 3.1 kg/cm2, were included in the final analysis (Table 1). 34% of the SPK recipients were > = 55 years old. The sex was distributed equally between the two age groups, with 85.7% males in the elderly group and 89.7% males in the younger group (Table 1). Furthermore, no difference was observed between the two groups in terms of BMI, dialysis rate, PRA, cold ischemia time of renal graft, comorbidities of cardiovascular events and cerebrovascular diseases before transplantation (Table 1). Parameters about donor’s, including age, sex and BMI were indifferent between the two groups (Table 1).

**Table 1:** Baseline characteristics

Variables	level	Overall	>=55	< 55	*p*
n		103	35	68	
**Recipients**					
Sex, n (%)	F	12 (11.7)	5 ( 14.3)	7 ( 10.3)	0.55
	M	91 (88.3)	30 ( 85.7)	61 ( 89.7)	
Age, years (mean (SD))		50.26 (9.16)	59.95 (4.10)	45.27 (6.71)	< 0.001
BMI, kg/cm^2^ (mean (SD))		24.68 (3.05)	24.73 (2.52)	24.65 (3.31)	0.906
Dialysis, n (%)	No	13 (12.6)	5 ( 14.3)	8 ( 11.8)	0.715
	Yes	90 (87.4)	30 ( 85.7)	60 ( 88.2)	
DM duration,, years (mean (SD))		19.12 (10.46)	20.04 (8.94)	18.64 (11.19)	0.522
CVD_before_transplantation, n (%)	Yes	30 (29.1)	14 ( 40.0)	16 ( 23.5)	0.081
	No	73 (70.9)	21 ( 60.0)	52 ( 76.5)	
CRD_before_transplantation, n (%)	Yes	14 (13.6)	4 ( 11.4)	10 ( 14.7)	0.646
	No	89 (86.4)	31 ( 88.6)	58 ( 85.3)	
PRA, n (%)	No	92 (89.3)	32 ( 91.4)	60 ( 88.2)	0.619
	Yes	11 (10.7)	3 ( 8.6)	8 ( 11.8)	
Induction, n (%)	Anti-IL-2R	5 ( 4.9)	2 ( 5.7)	3 ( 4.4)	0.771
	rATG	98 (95.1)	33 ( 94.3)	65 ( 95.6)	
CNI, n (%)	Tac	77 (82.8)	26 ( 83.9)	51 ( 82.3)	0.846
	CsA	16 (17.2)	5 ( 16.1)	11 ( 17.7)	
Antiproliferative_drugs, n (%)	EC-MPS	47 (52.8)	15 ( 50.0)	32 ( 54.2)	0.208
	Mizoribine	6 ( 6.7)	4 ( 13.3)	2 ( 3.4)	
	MMF	36 (40.4)	11 ( 36.7)	25 ( 42.4)	
C-peptide, ng/mL		2.35 (0.97)	2.36 (1.00)	2.34 (0.97)	0.957
Followuptime, years (mean (SD))		4.05 (1.43)	3.96 (1.61)	4.10 (1.34)	0.65
**Donors**					
Donor_age, years (mean (SD))		30.18 (13.62)	31.15 (11.64)	29.61 (14.74)	0.608
Donor_Sex, n (%)	F	17 (16.5)	3 ( 8.6)	14 ( 20.6)	0.12
	M	86 (83.5)	32 ( 91.4)	54 ( 79.4)	
Donor_BMI, kg/cm^2^ (mean (SD))		27.87 (12.87)	31.87 (9.25)	25.94 (14.02)	0.145
KidCIT, minute (mean (SD))		4.12 (1.18)	4.50 (1.24)	3.97 (1.13)	0.091

BMI: Body Mass Index; CVD: cardiovascular disease; CRD: cerebrovascular disease; PRA ( / ), if the percent panel-reactive antibody 10%, then the PRA is defined as positive, otherwise is negative; Anti-IL-2R, interleukin 2 receptor; rATG, anti-thymocyte globulin; CNI, calcineurin inhibitor; CsA, cyclosporine A; EC-MPS, enteric-coated mycophenolate sodium; MMF, mycophenolate mofetil; op, operation; Tac, tacrolimus; KidCIT, cold ischemic time of kidney graft; SD, standard deviation;

### Immunosuppression

About the induction regime and immunosuppression maintaining strategy, 95.1% (98) of the recipients were induced with rATG, Tac was administrated in 82.6% of recipients and MPA was in 93.3% patients (Table 1). The proportion of immunosuppression strategy was similar between the < 55 group and > = 55 group (Table 1).

### Patient survival and grafts cumulative incidence

**Fig. 1:**
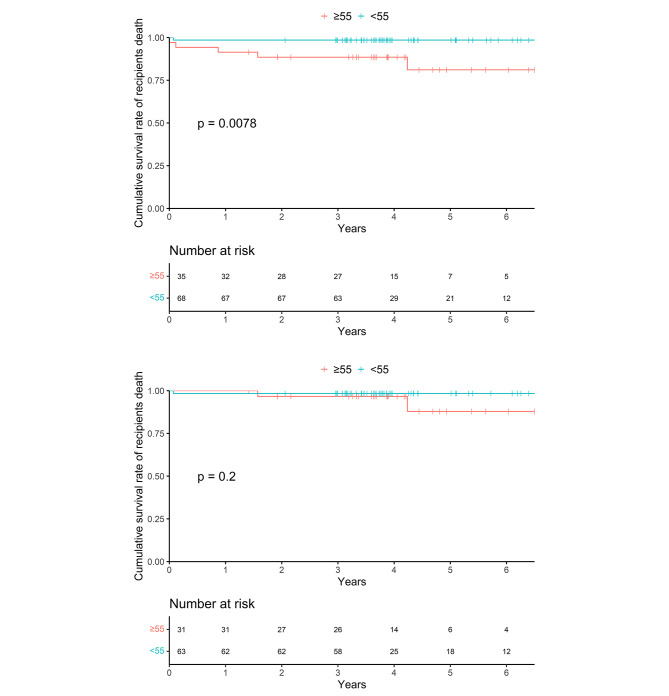
(**a**) Kaplan-Meier curve of patient survival. (**b**) Kaplan-Meier curve of patient survival with G+

During the follow-up period, 6 recipients (5.8%) died, and the fraction of patient death was significantly higher in older group (14.3% vs. 1.5%, Chi-square *p* = 0.009) (Table 2). Among the 6 deaths, 2 died from infection, 2 due to myocardial infarction, and 1 was because of cerebral hemorrhage. Figure 1 shows Kaplan-Meier curves of recipients. The 5-year patient survival rate of the > = 55 group was significantly lower than that of < 55 group (81.1% vs. 98.5%, log-rank *p* = 0.0078) (Table 3; Fig. 1a). Albeit, for patients with grafts (either kidney graft or pancreas graft) functioning during perioperative period, the recipient survival rate was comparable (87.9% of > = 55 group vs. 98.4% of < 55 group, log-rank *p* = 0.2) (Table 3; Fig. 1b).

**Table 2:** Complications and survival outcomes

Variables	level	Overall	>=55	< 55	*p*	
n		103	35	68		
Kidney Rejection, n(%)	Yes	26 (25.2)	10 ( 28.6)	16 ( 23.5)	0.577	
	No	77 (74.8)	25 ( 71.4)	52 ( 76.5)		
Pancreas Rejection, n(%)	Yes	6 (5.83)	2 (5.71)	4 (5.88) 0.999		
	No	97 (94.17)	33 (94.29)	64 (94.12)		
Readministration, n(%)	Yes	20 (19.4)	10 ( 28.6)	10 ( 14.7)	0.092	
	No	83 (80.6)	25 ( 71.4)	58 ( 85.3)		
Infection, n(%)	Yes	34 (33.0)	11 ( 31.4)	23 ( 33.8)	0.807	
	No	69 (67.0)	24 ( 68.6)	45 ( 66.2)		
Reoperation, n(%)	Yes	10 ( 9.7)	5 ( 14.3)	5 (7.4)	0.26	
	No	93 (90.3)	30 ( 85.7)	63 (92.6)		
DGF, n(%)	Yes	1 ( 1.0)	0 ( 0.0)	1 (1.5)	0.471	
	No	102 (99.0)	35 (100.0)	67 ( 98.5)		
CVD_after_transplantation, n(%)	Yes	32 (31.1)	12 ( 34.3)	20 (29.4)	0.613	
	No	71 (68.9)	23 ( 65.7)	48 (70.6)		
CRD_after_transplantation, n(%)	Yes	6 ( 5.8)	2 ( 5.7)	4 ( 5.9)	0.972	
	No	97 (94.2)	33 ( 94.3)	64 (94.1)		
Patient_death, n(%)	Yes	6 ( 5.8)	5 ( 14.3)	1 ( 1.5)	0.009	
	No	97 (94.2)	30 ( 85.7)	67 (98.5)		
Cause of patient death, n(%)						
Infection		2	2(40%)(	-	0.999	
myocardial infarction		3	2(40%)	1(100%)		
cerebral hemorrhage		1	1(20%)	-		
Pancreas_graft_loss n(%)	Yes	14 (13.6)	7 ( 20.0)	7 ( 10.3)	0.173	
	No	89 (86.4)	28 ( 80.0)	61 ( 89.7)		
Kidney_graft_loss, n(%)	Yes	10 ( 9.7)	6 ( 17.1)	4 ( 5.9)	0.068	
	No	93 (90.3)	29 ( 82.9)	64 ( 94.1)		
Pancreas_graft_loss_CR, n(%)	Graft_loss	10 ( 9.7)	4 ( 11.4)	6 ( 8.8)	0.181	
	Death with functioning graft	4 ( 3.9)	3 ( 8.6)	1 ( 1.5)		
	Functioning	89 (86.4)	28 ( 80.0)	61 ( 89.7)		
Kidney_graft_loss_CR, n(%)	Graft_loss	9 ( 8.7)	5 ( 14.3)	4 ( 5.9)	0.127	
	Death with functioning graft	1 ( 1.0)	1 ( 2.9)	0 ( 0.0)		
	Functioning	93 (90.3)	29 ( 82.9)	64 ( 94.1)		

DGF: delayed graft function; CVD: cardiovascular disease; CRD: cerebrovascular disease; CR: competing risk model

**Table 3:** Survival rates

Survival rate	Overall	>=55	< 55	*p*	test
Patient					
5-year survival rate	92.8%	81.1%	98.5%	0.0078	log-rank
5-year survival rate(G+)	95.1%	87.9%	98.4%	0.2	log-rank
Kidney graft					
5-year survival rate	87.6%	82.4%	90.8%	0.055	log-rank
5-year cumulative incidence of graft loss	10.3%	14.7%	7.7%	0.064	Grey’s test
5-year cumulative incidence of graft loss(G+)	8.5%	10.2%	7.3%	0.156	Grey’s test
Pancreas graft					
5-year survival rate	80.2%	70.3%	86.1%	0.17	log-rank
5-year cumulative incidence of graft loss	14.4%	18.6%	12.4%	0.673	Grey’s test
5-year cumulative incidence of graft loss(G+)	9.4%	14.5%	7.0%	0.466	Grey’s test

**Fig. 2:**
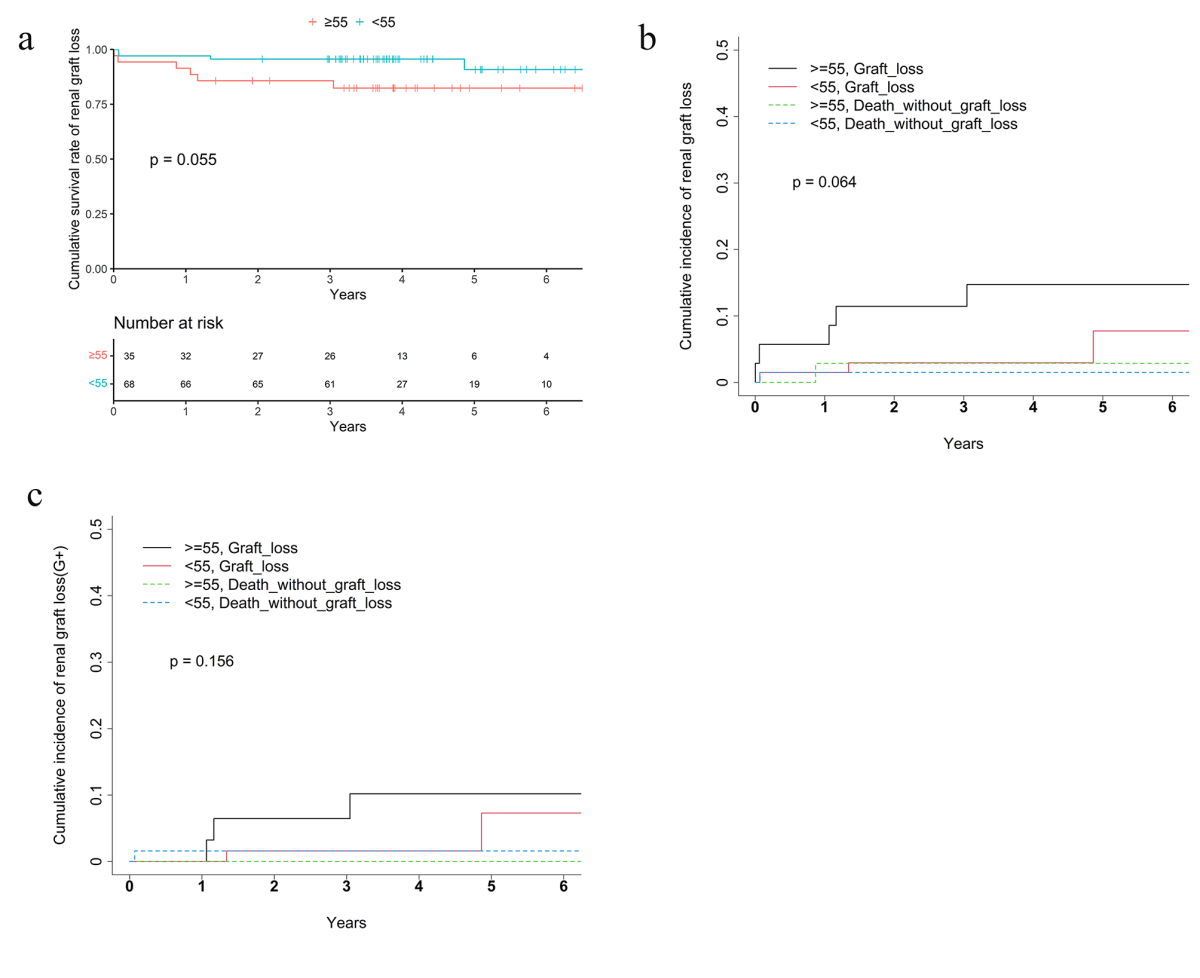
(**a**) Kaplan-Meier curve of kidney graft survival. (**b**) Competing risk analysis of kidney graft loss. (**c**) Competing risk analysis of kidney graft loss with G+

**Fig. 3:**
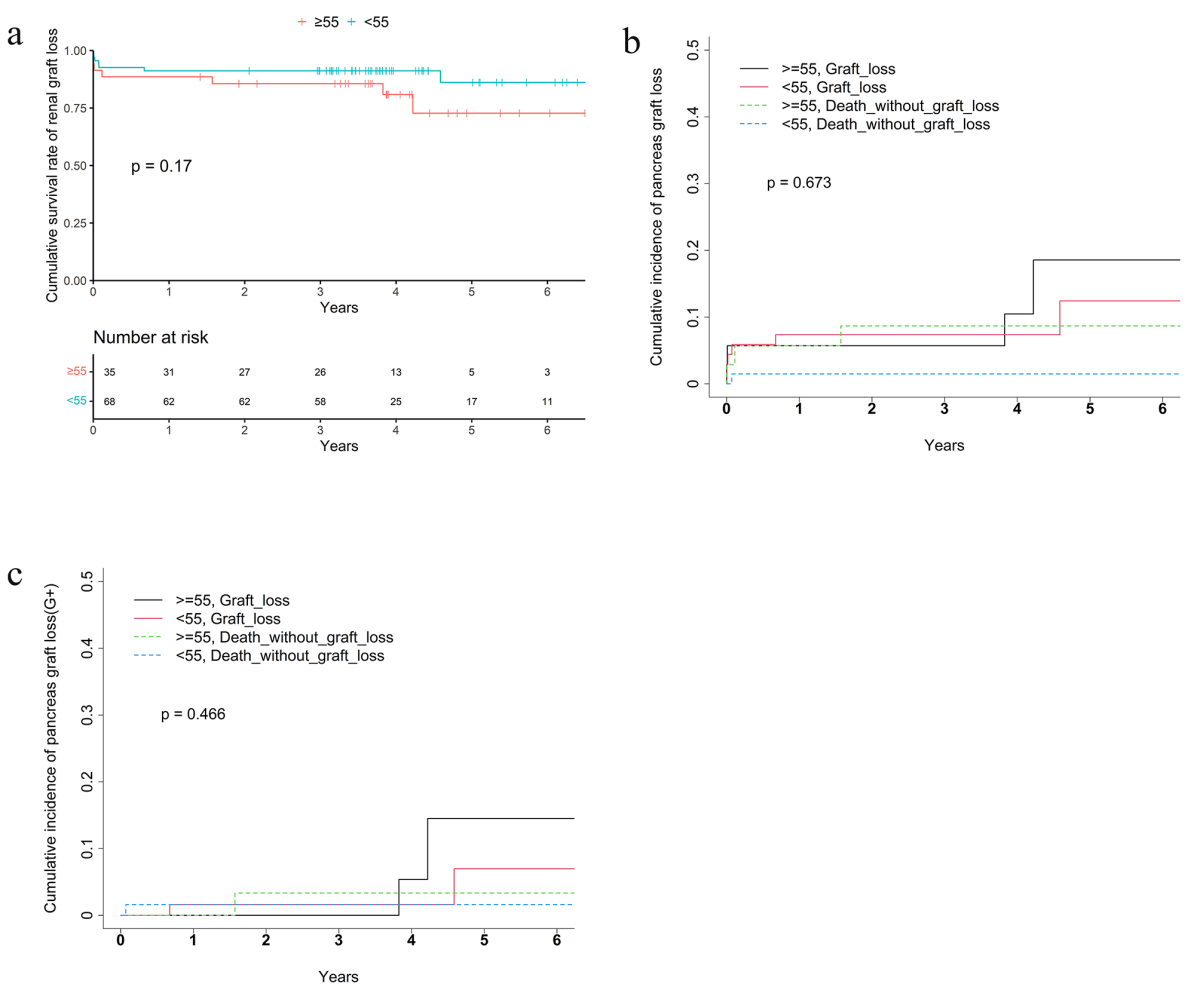
(**a**) Kaplan-Meier curve of pancreas graft survival. (**b**) Competing risk analysis of pancreas graft loss (**c**) Competing risk analysis of pancreas graft loss with G+

G+: for patients with both grafts functioning during perioperative period;

The overall kidney graft survival was lower in the elderly group as compared to younger group 82.4% vs. 90.8% respectively. After taking into account the competing risk of death, the 5-year cumulative incidence of graft failure was comparable between different age groups (for renal graft, 14.7% of > = 55 group vs. 7.7% 189 of < 55 group, Grey’s test *p* = 0.064; for pancreas graft, 18.6% vs. 12.4% for the > = 55 group and < 55 group separately, Grey’s test *p* = 0.683) (Table 3; Figs. 2b and 3b).

### Complications and metabolic outcomes

**Fig. 4:**
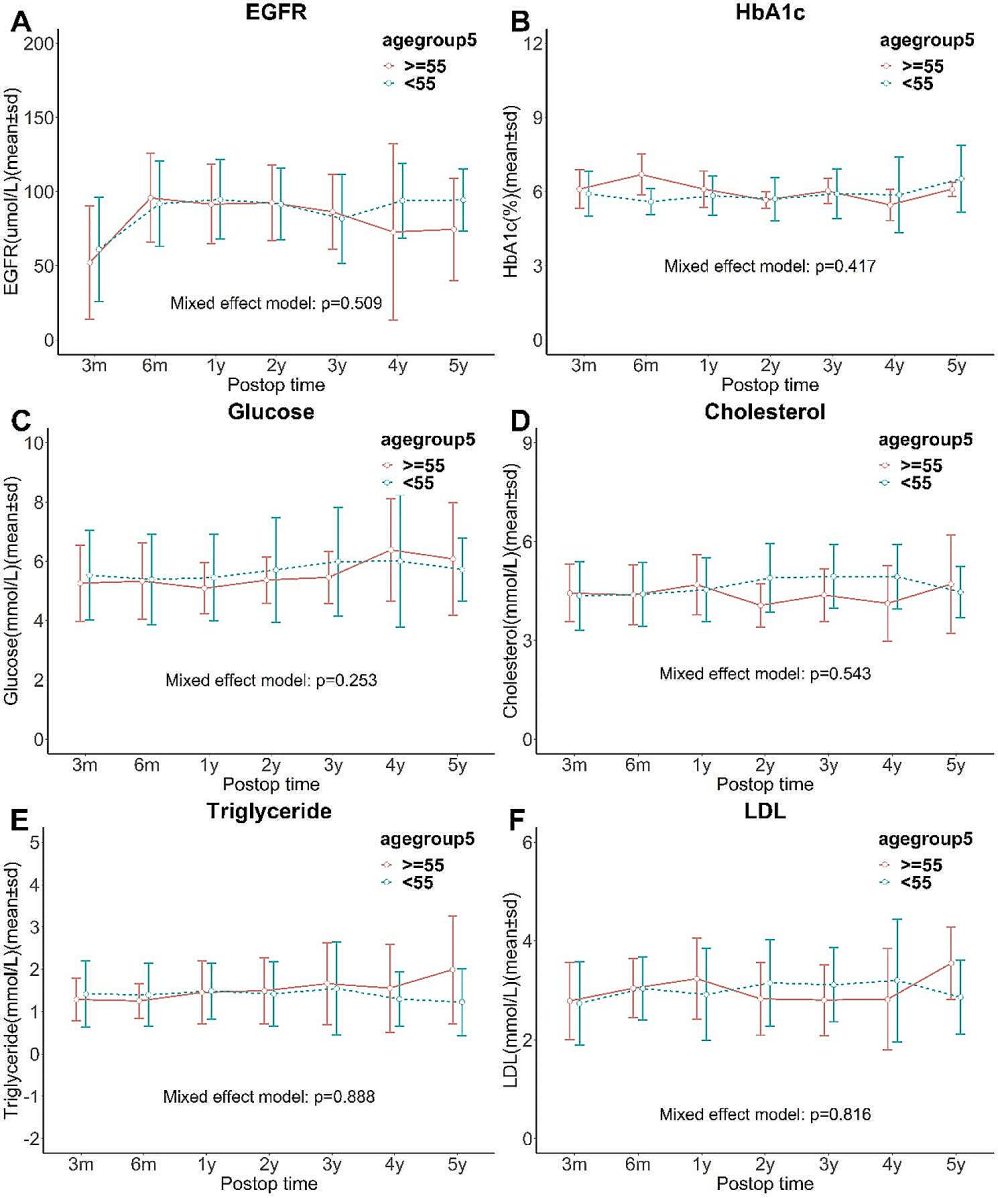
Comparison of metabolic outcomes between two groups

Regarding post transplantation complications, the rate of graft rejection was comparable between two groups (28.6% of ≥ 55 group vs. 23.5% of < 55 group, *P* = 0.577; 5.7% of ≥ 55 group vs. 5.9% of < 55 group, *P* = 0.999) (Table 2). The rates of DGF, re-administration, reoperation, cardiovascular diseases after transplantation, cerebrovascular diseases after transplantation, infection were not significantly different (Table 2). The level of HbA1c, fasting blood glucose, triglyceride, cholesterol, low-density lipoprotein, and eGFR were comparable between the two groups as well (Fig. 4).

## Discussion

The objective of this retrospective cohort study was to analyze the influence of recipient age on the clinical outcomes of T2DM SPK recipients. Our results indicated that the elderly recipients had inferior overall 5-year survival outcomes and most of them died more from infection and cardiovascular diseases. For those with functioning grafts during perioperative period, the 5-year survival rate was indifferent. Concerning the graft survival outcomes, by taking into account the competing risk in estimating probabilities of graft loss, the cumulative incidence of graft loss was indifferent. Additionally, no relationship between age and post-SPK complications or metabolic outcomes was found.

Recipient age and its influence on clinical outcome after pancreas transplantation among T1DM recipients has already been studied and widely diverging results regarding post-transplant outcomes were reported. Several published studies [7, 15, 16, 30, 31] demonstrated a correlation between recipient age and patient survival. A latest study by Messner et al. [30] reported that the old recipients had a significantly inferior patient survival compared to young recipients, which was in line with our results. Similarly, Gurung et al. [7] demonstrated that the survival rate was inferior in the age group of ≥ 50 years recipients (*p* = 0.013) and Mittal et al. [31] found that the elderly recipients had a lower rate of survival. One of the largest studies on this topic was reported by Siskind et al. [16]. They included 20,854 patients from the UNOS database between 1996 and 2012 and divided patients into different groups age categories and found that patients’ survival significantly dropped with increasing age [30]. Likewise, Freise et al. [15] reported increased morbidity and mortality of elder SPK recipients. By contrast, Schenker et al. [13] reported comparable survival rates of patient, pancreas, and kidney graft among euro-transplant recipients. Additionally, six single-center studies also reported comparable patient and graft survival in elderly recipients [10–12, 32]. In our cohort, the overall patients’ survival outcomes were lower in the younger group. For the subgroup of patients with functioning graft during peri-operative period, the long-term mortality was equivalent between the two age groups. Moreover, The analogous progression of metabolic profiles substantiated that there was no significant difference in the clinical outcomes between the elderly cohort and the younger cohort. Also, the comparable rates of re-administration, reoperation and infection after transplantation suggested the efficacy and safety for the elder SPK recipients.

To date, this is the first large single-center study evaluating the outcomes of SPK in T2DM ESRD patients older than 55 years with comparable risks for death or graft loss than in younger recipients. The majority of the SPK recipients were T1DM in western countries. Even though the number of T2DM recipients increased remarkably since 2016 according to the 2019 OPTN pancreas transplantation report, T2DM recipients accounted for 22.4% of SPK and T1DM for 74% [9]. By contrast in mainland China, mostly were T2DM recipients, accounting for more than 70% of total SPK recipients [33, 34]. According to previous reports, T2DM SPK recipients had longer duration of pretransplant dialysis, higher rates of diabetes related comorbidities, like arterial obstructive disease, retinopathy and neuropathy [18, 19, 35]. In specific, there’s a direct correlation between cardiovascular disease and age [36]. It has been noted in the past that older individuals who have undergone pancreas transplants have a higher incidence of significant adverse cardiovascular disease compared to their younger counterparts. For instance, up to 64% of those over 55 years old required cardiac catheterization, compared to 32% of those under 34 years old [11].. In a study conducted by Afaneh et al. [37], it was found that coronary artery disease was present in 18% of the group aged below 50 years and 47% of the group aged 55 years and above. Additionally, Laurence et al. [32] reported that a larger percentage of recipients aged 55 years and above underwent preoperative cardiac intervention (46% vs. 13%). In our study, after a thorough pre-transplant assessment, the rates of complications related to pre-transplant diabetes were found to be similar in the group aged 55 and above and the group aged below 55.

This study is limited by its retrospective design and from a single center. The statistical analysis might be biased because there are relatively few patients in each group, particularly in the older age cohort. The age group cut-off points may seem arbitrary but they are based on previous studies related to this topic and hold clinical significance, as many centers use age as a determining factor when selecting recipients [38].. On the other hand, the strengths of the study lie in the detailed data collection and the use of a competing risk analysis for evaluating graft survival data.

## Conclusion

Consequently, there has been a rise in the number of older patients undergoing SPKT in recent years. The decision to proceed with SPKT is typically based on the severity of the disease, comorbidity, and probable benefits, rather than age alone. For T2DM recipients, SPKT may provide both a survival and quality of life benefit to all appropriately selected candidates even though those above 55 years old on the premise that the grafts survived the perioperative period.

## Data Availability

The data that support the findings of this study are available from the corresponding author, [Jie Zhao], upon reasonable request.

## Abbreviations

SPK: Simultaneous Kidney Pancreas Transplantation
KTA: Kidney Transplantation Alone
ESKD: End-Stage-Kidney-Disease
DM: Diabetes Mellitus
T2DM: Type 2 Diabetes Mellitus
BMI: Body Mass Index
PRA: Panel Reactive Antibody
SD: Standard Deviation
rATG: Anti-thymocyte globulin
Anti-IL-2R: Interleukin 2 Receptor
CNI: Calcineurin inhibitor
Tac: Tacrolimus
CsA: Cyclosporine A
MMF: Mycophenolate mofetil
EC-MPS: Enteric-coated mycophenolate sodium
CVD: Cardiovascular diseases
CRD: Cerebrovascular diseases
DGF: Delayed graft function
HbA1c: Glycated hemoglobin test
LDL: Low density lipoprotein
HDL: High density level
